# Total arch replacement with frozen elephant trunk for distal arch aneurysm with aberrant right subclavian artery after type B aortic dissection

**DOI:** 10.1016/j.xjse.2025.100070

**Published:** 2025-08-25

**Authors:** Yuki Akaguma, Hideki Tsubota, Masanori Honda, Masafumi Kudo, Hitoshi Okabayashi

**Affiliations:** Department of Cardiovascular Surgery, Mitsubishi Kyoto Hospital, Kyoto, Japan

**Figure:**
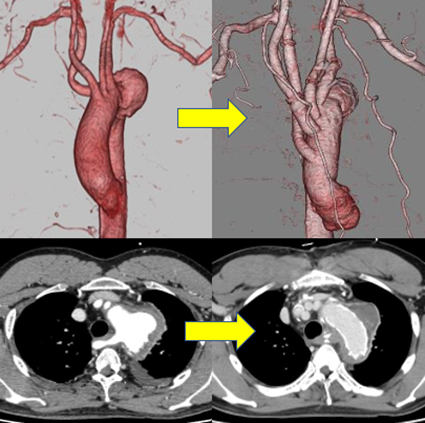
TAR + FET with ligation at the origin of the ARSA.

**Central Message**

We report a successful case of total arch replacement with frozen elephant trunk accompanied by ligation at the origin of an aberrant right subclavian artery.

Aberrant right subclavian artery (ARSA) is a congenital vascular anomaly occurring in 0.5% to 1.8% of the general population, frequently associated with aortic aneurysms.^1^ We describe a 70-year-old man who developed a rapidly enlarging aortic arch aneurysm involving ARSA after Stanford type B acute aortic dissection. The patient successfully underwent total arch replacement plus frozen elephant trunk (FET) with ligation at the origin of the ARSA. This research was approved by the Ethics Committee of Mitsubishi Kyoto Hospital (approval no. 25-4, on April 17, 2025). Written informed consent for publication of this case report was obtained from the patient.

## Case Report

**Figure 1:**
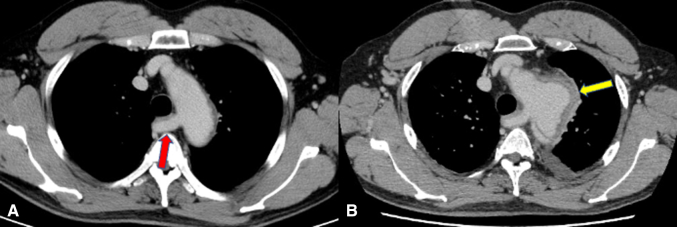
A, Computed tomography scan 3 weeks before presentation revealed an aberrant right subclavian artery (*red arrow*). B, Rapid expansion of the aneurysm to 58 mm (*yellow arrow*).

A 70-year-old man was transferred to our hospital with a 10-day history of back pain and lower limb claudication. He had undergone surgery for cecal cancer at age 65 years. A previous computed tomography (CT) scan had revealed an ARSA with a 38-mm aortic arch aneurysm (Figure 1, *A*). On admission, CT scanning showed type B acute aortic dissection with the aneurysm, which had rapidly enlarged to 58 mm (Figure 1, *B*). The ankle-brachial index was decreased, and acute kidney injury was present, indicating semiemergent surgery necessary.

Cardiopulmonary bypass was initiated via ascending aortic and right atrial cannulation after median sternotomy. After systemic cooling to 28 °C, the aorta was crossclamped and cardiac arrest was achieved with antegrade cardioplegia. Under moderate hypothermic circulatory arrest, selective antegrade cerebral perfusion was started at 800 mL/min (10 mL/kg/min) via both common carotid arteries and the left subclavian artery while monitoring to ensure no decline in cerebral oxygen saturation. An intimal tear was just distal to the origin of ARSA. The ARSA origin was ligated with 3-0 polypropylene sutures. A 27-mm × 12-cm Frozenix stent graft (Japan Lifeline Inc) was deployed in zone 2. A 26-mm, 4-branched graft was anastomosed using the turn-up technique.^2^ After reconstruction of the left subclavian artery, the proximal anastomosis was performed first to minimize the aortic crossclamp time, followed by anastomoses of the graft branches to the bilateral common carotid arteries. The ARSA was ligated, transected, and anastomosed to a graft branch routed beneath the innominate vein. The patient was weaned from bypass without difficulty (Video 1).

**Figure 2:**
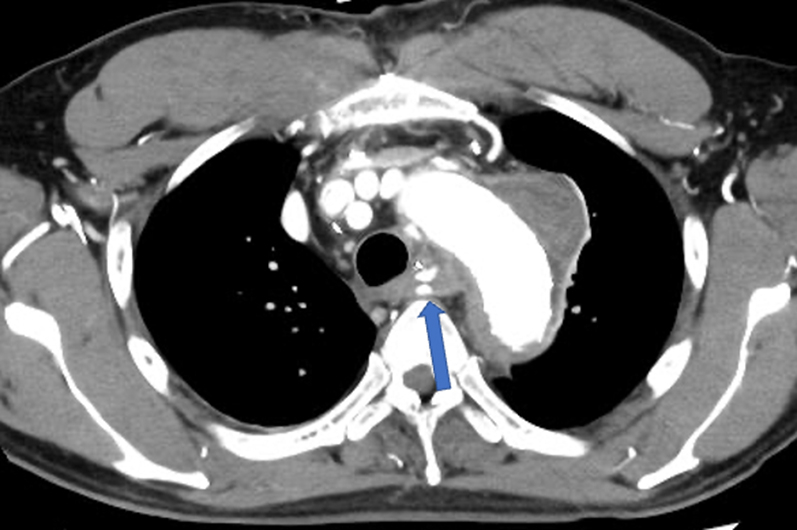
Postoperative computed tomography scan showed aneurysm thrombosis and ligation of the aberrant right subclavian artery reinforced with felt pledgets (*arrow*).

Postoperatively, claudication and renal function improved, and no neurological deficits were noted except for dysphagia and hoarseness. Laryngoscopy revealed incomplete paralysis of the right vocal cord; however, nearly complete recovery was observed at 4 months postoperatively, with no significant impairment in daily activities. CT confirmed aneurysm thrombosis and residual flow in the false lumen below the diaphragm due to a distal reentry (Figure 2, Video 1).

## Discussion

ARSA is a congenital vascular anomaly and associated with thoracic aortic aneurysms.^1^ In this case, surgery was indicated on the basis of 2 findings: (1) rapid enlargement of the aortic aneurysm and (2) signs of malperfusion. In this case, because of the patient's relatively young age, anatomical reconstruction was prioritized, and total arch replacement plus FET was performed with mediastinal reconstruction of the right subclavian artery. In patients with an ARSA, the right laryngeal nerve does not loop around the right subclavian artery but rather branches directly from the vagus nerve at the level of the thyroid gland—a variant known as the nonrecurrent inferior laryngeal nerve.^3^, ^4^, ^5^ Therefore, mediastinal reconstruction of the ARSA does not increase the risk of nerve injury. During mediastinal reconstruction of the ARSA, ligation solely at the anastomotic site may leave branches between the origin and the anastomosis, which could lead to aneurysmal enlargement in the long term even if the origin of the ARSA is covered with FET. To mitigate this risk, we performed ligation at the ARSA origin. This approach eliminates the risk of retrograde flow and allows for definitive single-stage repair. It is particularly useful in younger patients with shallow chests, where access to the ARSA origin is technically feasible.

## Conflict of Interest Statement

The authors reported no conflicts of interest.

The *Journal* policy requires editors and reviewers to disclose conflicts of interest and to decline handling or reviewing manuscripts for which they may have a conflict of interest. The editors and reviewers of this article have no conflicts of interest.

## Supplementary Data

Video 1 Demonstrating TAR + FET with ligation at the origin of an ARSA. Video available at: https://www.jtcvs.org/article/S2950-6050(25)00029-4fulltext.
