# Supplementary figures and images for: Total arch replacement with frozen elephant trunk for distal arch aneurysm with aberrant right subclavian artery after type B aortic dissection
Source: JTCVS Struct Endovasc. 2025 Aug 25;8:100070. doi: 10.1016/j.xjse.2025.100070 (PMC13244726; doi:10.1016/j.xjse.2025.100070)

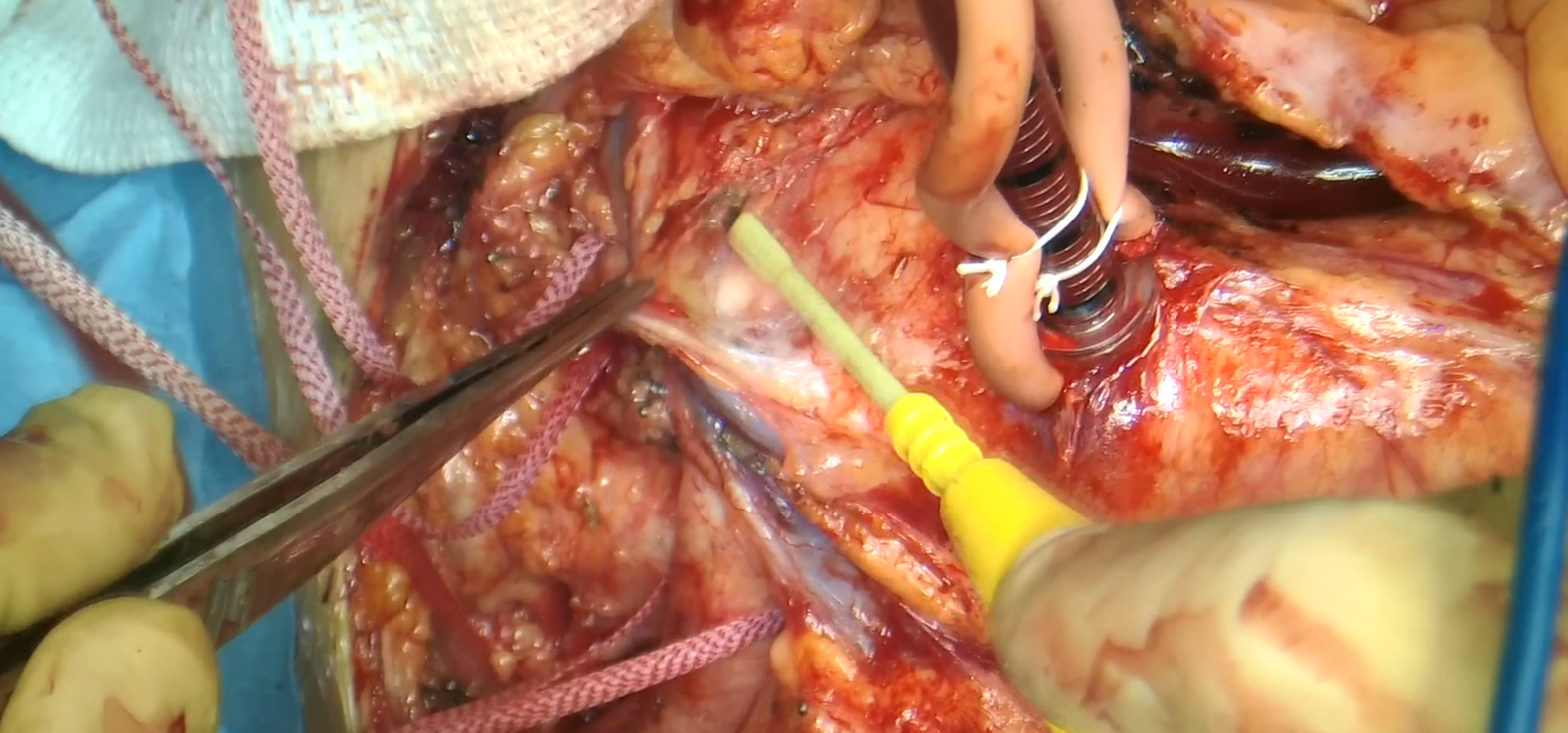

Supplement: Video 1 — Demonstrating TAR + FET with ligation at the origin of an ARSA. Video available at: https://www.jtcvs.org/article/S2950-6050(25)00029-4fulltext. [file fx2.jpg]
